# Identification of Human Retinal Organoid Cell Differentiation-Related Genes via Single-Cell Sequencing Data Analysis

**DOI:** 10.1155/2022/9717599

**Published:** 2022-08-08

**Authors:** He Dong, Liang Yu, Jian Song, Lili Ji, Xiaoxia Yu, Lijun Zhang

**Affiliations:** ^1^Department of Ophthalmology, Dalian No. 3 People's Hospital, Dalian 116033, China; ^2^Department of Blood Purification, Affiliated Zhongshan Hospital of Dalian University, Dalian 116001, China

## Abstract

**Objective:**

To study the development process of the human retina, we analyzed the development track of main cell types and transitional cell populations, identifying the retinal organoid cell differentiation-related genes (RDRGs).

**Methods:**

Single-cell RNA sequencing data (scRNA-Seq) of human retinal organoids were downloaded from Gene Expression Omnibus (GEO) database in this study. Data were processed with quality analysis and analysis of variance. Principal component analysis and *t*-distributed stochastic neighbor embedding were used to conduct dimension reduction analysis and type annotation for the screened data. Marker genes and RDRGs were identified by differential analysis. Cell differentiation characteristics were determined by trajectory analysis. Enrichment pathways were analyzed by Gene Ontology(GO) and Kyoto Encyclopedia of Genes and Genomes(KEGG), and functional modules were obtained by protein-protein interaction (PPI) network analysis.

**Results:**

iPSCs were mainly located at the root of differentiation trajectory, while neurons and astrocytes were distributed in different branches, respectively. Meanwhile, 220 RDRGs were obtained. They were involved in the biological functions related to vision and visual development, as well as significantly enriched in signaling pathways associated with retinal vascular development and retinal neuroregulation. Protein-protein interaction network construction and functional subnetwork analysis were conducted on RDRGs, and two functional submodules were obtained. The enrichment analysis presented that the two submodules played a vital role in retinal development, visual perception, and cell respiration.

**Conclusions:**

This study identified RDRGs and revealed the biological functions involved in these genes, which are expected to provide evidence for researching retinal development and diseases.

## 1. Introduction

As the premise for vision, vertebrate retina is a thin layer of cells in the posterior lining of the eyeball. The retina is transformed from the front of the brain by a complex network of signals. At present, the human retina is mainly studied from the perspective of the organoid derived from stem cells [1]. Stem cells include human embryonic stem cells (hESCs) and induced pluripotent stem cells (hiPSCs). These two types of cells can differentiate into the precursors of retinal photoreceptor cells (cones and rods) [2]. Retinal differentiation methods have also evolved from 2D adherent cultures to 3D cultures that can more easily replicate development in humans [1]. For example, Zhong et al. [3] rebuilt a complete tissue structure of retina via hiPSCs. Wahlin et al. [4] derived 3D microretinas from human pluripotent stem cells (hPSCs), which were more similar to the retina structure observed in vivo. The study of retinal organoid differentiation in vitro is of great significance to the cognition of human retinal diseases.

Abnormal gene expression could cause retinal degeneration. A previous study presented that in a mouse model of oxidative retinal degeneration, TMEM97 knockout exacerbated oxidative stress response, thereby aggravating oxidative retinal degeneration [5]. It was also elaborated that increasing HTRA1 expression played a role in AMD [6]. With the popularization of high-throughput sequencing technology in the last few years, accumulating research has applied single-cell RNA sequencing (scRNA-Seq) technology into the analysis of disease transcriptomics. Compared with bulk RNA-Seq technology, scRNA-Seq shows superiority in revealing gene expression features of different cells and in studying cell differentiation trajectory [7]. Wang et al. [8] identified differentially expressed genes related to glioblastoma (GBM) cell differentiation via analyzing scRNA-Seq data of human GBM. Voigt et al. [9] analyzed scRNA-Seq data of choroid in human retinal pigment epithelium (RPE), revealing the gene expression characteristics of choroidal cell population, and pointed out that the upregulation of RGCC expression might be related to the occurrence of AMD [9].

scRNA-Seq data of human retinal organoids were downloaded from Gene Expression Omnibus (GEO) database and analyzed through a series of bioinformatics methods. Cell subpopulation was identified during differentiation of human retinal organoids through trajectory analysis, and retinal organoid cell differentiation-related genes (RDRGs) were also identified. Enrichment analysis exhibited that these genes were mainly significantly enriched in biological functions related to visual development and retinal neuroregulation-relative signaling pathways. This study is expected to provide a strong theoretic basis for retinal development and disease research.

## 2. Materials and Methods

### 2.1. Data Sources and Technical Lines

**Figure 1:**
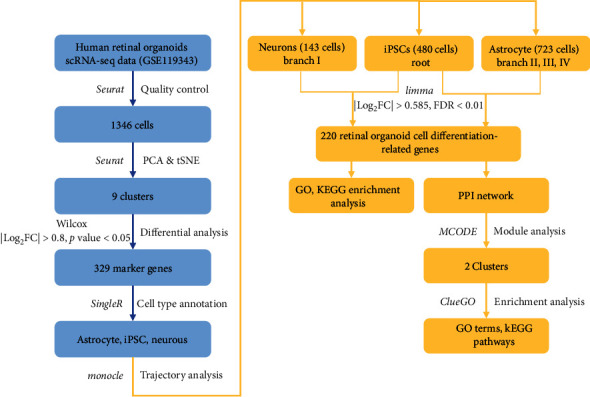
Technical route of scRNA-Seq data analysis of retinal organoids. Blue refers to dimension reduction clustering and cell annotation process of single cell sequencing data. Yellow refers to RDRG recognition and function analysis.

scRNA-Seq data (GSE119343) of human retinal organoids including cone cells were downloaded from GEO database (https://www.ncbi.nlm.nih.gov/geo/). Samples were retinal organs with 8 months of development. scRNA-Seq data included sequencing data of 1346 cells from 72 retinal organoids [10] (Data S1). The sequencing data were based on Illumina HiSeq 2500 (Homo sapiens) sequencing platform (GPL16791). The flowchart of this study was shown in Figure 1.

### 2.2. Processing of scRNA-Seq Data

Quality analysis was performed on scRNA-Seq data by using the R package “Seurat” (https://github.com/satijalab/seurat) to delete low-quality sequencing data. Data exclusion criteria were as follows: (1) genes that can be detected in <10 cells, (2) cells with total gene number < 100, and (3) cells with mitochondrial expressed genes ≥ 10%. The preserved data were normalized by the LogNormalize method (https://rdrr.io/github/LTLA/scuttle/man/logNormCounts.html).

Seurat is an R package for quality control, analysis, and exploration of scRNA-Seq data. Seurat enables users to identify and reveal sources of heterogeneity from the single-cell transcript side and integrate various types of single-cell data to undertake variance heterogeneity analysis and recognize HVG parameter as scRNA, selection method = ^“^VST^”^ [11].

### 2.3. Dimension Reduction Analysis and Cell Type Annotation

Principal component analysis (PCA) was performed on the normalized data with the RunPCA function in “Seurat” package. PC with *p* < 0.05 was chosen through JackStraw analysis and ElbowPlot for the following analysis. Then, cell clustering analysis was conducted on data via the nonlinear dimension reduction method of *t*-distributed stochastic neighbor embedding (tSNE) [12]. Differential analysis (∣log_2_FC | >0.8, *p* value < 0.05) was undertaken on different clusters with Wilcox test (https://www.rdocumentation.org/packages/stats/versions/3.6.2/topics/wilcox.test) to identify marker genes of cells in each cluster. Cell annotation information HumanPrimaryCellAtlasData in R package “SingleR” was used to annotate cells (https://www.bioconductor.org/packages/release/bioc/html/SingleR.html).

### 2.4. Trajectory Analysis and RDRG Identification

Trajectory analysis was conducted on cells using R package “monocle” to identify differentiation characteristics of cells. Monocle2 algorithm constructs single-cell differentiation trajectories based on reversed graph embedding (RGE) unsupervised machine learning technique [13]. Firstly, high-dimensional scRNA-Seq data were projected into low-dimensional space by reducing dimension with RGE. Afterward, each cell was taken as a part of cell differentiation to learn gene expression changes. A tree structure with “root” and “branch” was obtained. Finally, cells were arranged in the trajectory of the tree structure in pseudotime order. After single-cell trajectory was obtained, gene differential expression analysis was performed on cells distributed in the root and in different branches (∣log_2_FC | >0.585, FDR < 0.01) with cells distributed in the root as the control. The obtained differential expression genes were defined as RDRGs.

To analyze biological functions and signaling pathways RDRGs involved, GO annotation and KEGG enrichment analyses (qvalue < 0.05) were conducted on RDRGs with R package “clusterProfiler” [14].

### 2.5. Construction of RDRG Protein-Protein Interaction (PPI) Network and Submodule Analysis

PPI network analysis was performed on RDRGs on STRING website (https://string-db.org/) (interaction score > 0.4) [15]. Clustering analysis was undertaken in PPI network by using MCODE [16] plug-in of Cytoscape software, and functional submodules were constructed. To analyze biological functions of functional submodules, GO and KEGG enrichment analyses were performed on functional modules obtained by clustering through ClueGO [17] plug-in (qvalue < 0.05).

## 3. Results

### 3.1. scRNA-Seq Data Preprocessing and PCA

**Figure 2:**
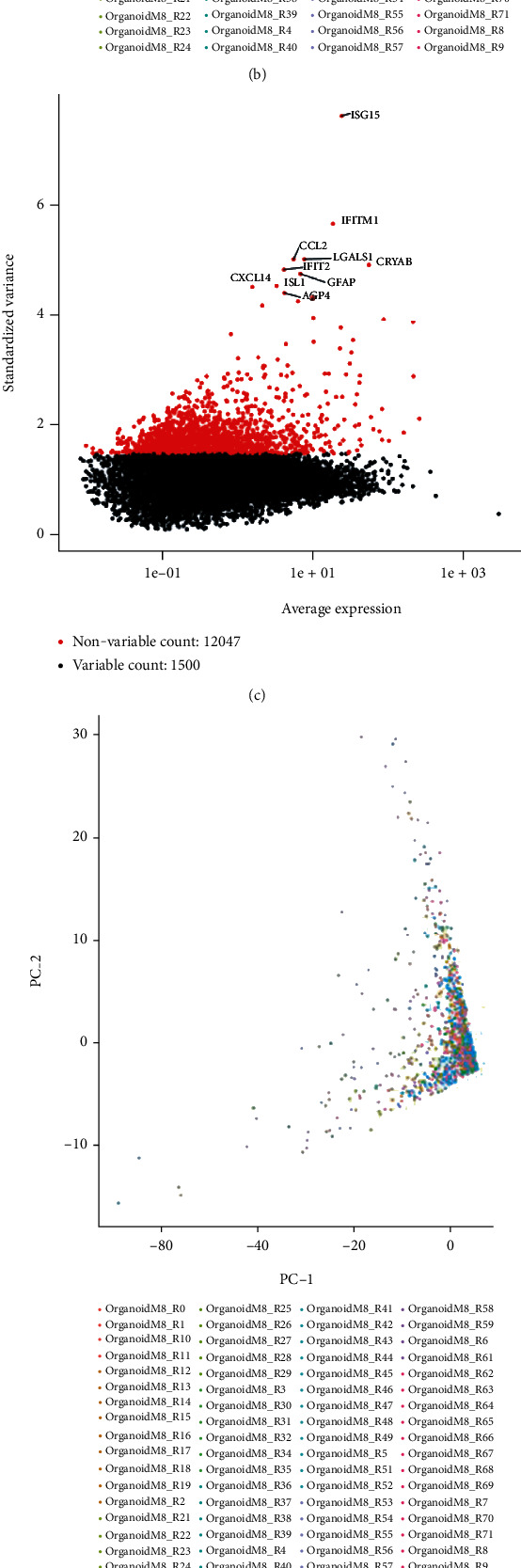
Quality analysis of scRNA-Seq data and PCA. (a) The left figure showed eliminated gene number of each cell data after quality analysis. The right figure showed total count number of each cell after quality analysis. (b) Correlation analysis between gene number detected by single cell and total count number obtained by sequencing. (c) Top 1,500 high variation genes obtained by variance analysis (red dots). (d) PCA plot. (e) PC with *p* < 0.05 through JackStraw analysis. (f) Standard deviation of PC shown in ElbowPlot.

Through quality analysis of scRNA-Seq data of human retinal organoids, 0 low-quality cells were eliminated, and 1,346 cell gene expression data from 72 retinal organoids were preserved (Figure 2(a)). Detected gene number was positively correlated with the total count number obtained from sequencing (Figure 2(b)). Variance analysis was undertaken on the 13,547 genes, and the top 1,500 high variation genes were acquired, among which top 9 genes were IFITM1, CCL2, LGALS1, CRYAB, IFIT2, GFAP, CXCL14, ISL1, and AQP4 (Figure 2(c)). PCA dimension reduction was performed on the top 1,500 high variation genes. As shown in Figure 2(d), PC_1 and PC_2 could not significantly separate cells derived from different retinal organoids. Lastly, 9 PCs were obtained through evaluation (*p* < 0.05) for the subsequent analyses (Figures 2(e) and 2(f)). Finally, we divided the cells of retinal organoids into 9 PCs.

### 3.2. Cell Type Annotation and Single-Cell Differentiation Trajectory Analysis

**Figure 3:**
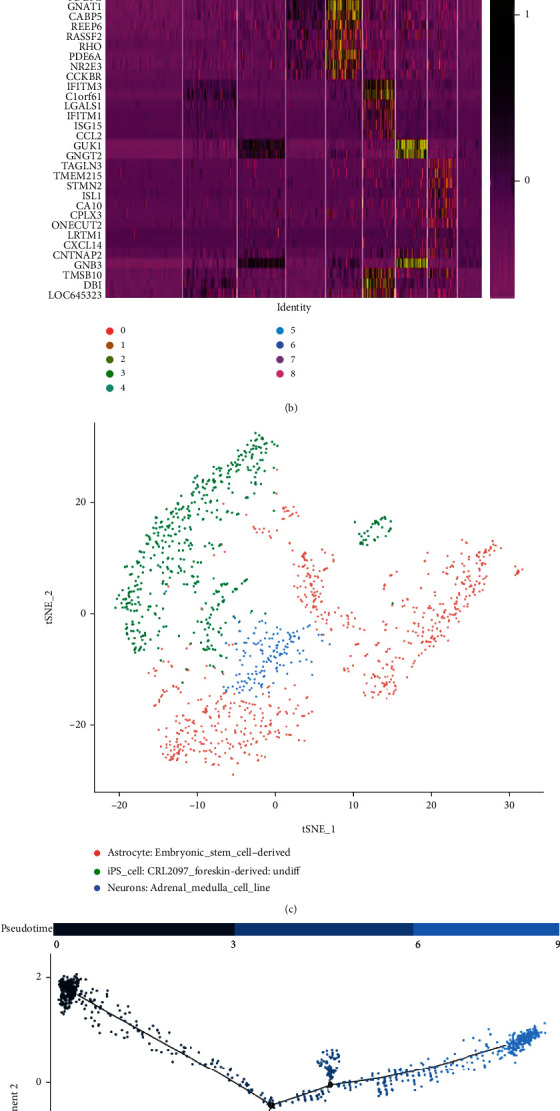
Cell annotation and trajectory analysis. (a) Nine clusters were obtained by cell clustering analysis through tSNE nonlinear dimension reduction. (b) Heat map of gene differential expression analysis of cells in each cluster (heat map only shows part of marker genes in each cluster of cells). (c) Cells in each cluster were annotated via marker genes to obtain iPSCs (green dots), neurons (blue dots), and astrocytes (pink dots). (d) Cells were sequenced along the trajectory according to pseudotime. Lighter color refers to higher differentiating level. (e) One root and four branches were obtained by trajectory analysis.

tSNE analysis was performed on 9 PCs obtained by linear dimension reduction, and cells were clustered into 9 clusters (Figure 3(a)). Differential analysis was conducted on different clusters to identify marker genes in each cell cluster (Figure 3(b)) (Data S1). A total of 3 types of cells were obtained by cell annotation with R package “SingleR.” Clusters 2, 6, 7, and 8 were annotated as iPSCs (*n* = 480). Cluster 3 including 143 cells was annotated as neurons. Clusters 0, 1, 4, and 5 including 723 cells were annotated as astrocytes (Figure 3(c)). Trajectory analysis was performed on annotated cells (Figures 3(d) and 3(e)). It was shown that iPSCs were mainly distributed in the root of trajectory. Neurons were mainly located in branch I. Astrocytes were mainly distributed in branch II, branch III, and branch IV. Together, through analysis of scRNA-Seq data of human retinal organoids, it was displayed that iPSCs could differentiate into astrocytes and neurons during the formation of human retinal organoids.

### 3.3. Identification of RDRGs and Enrichment Analyses

**Figure 4:**
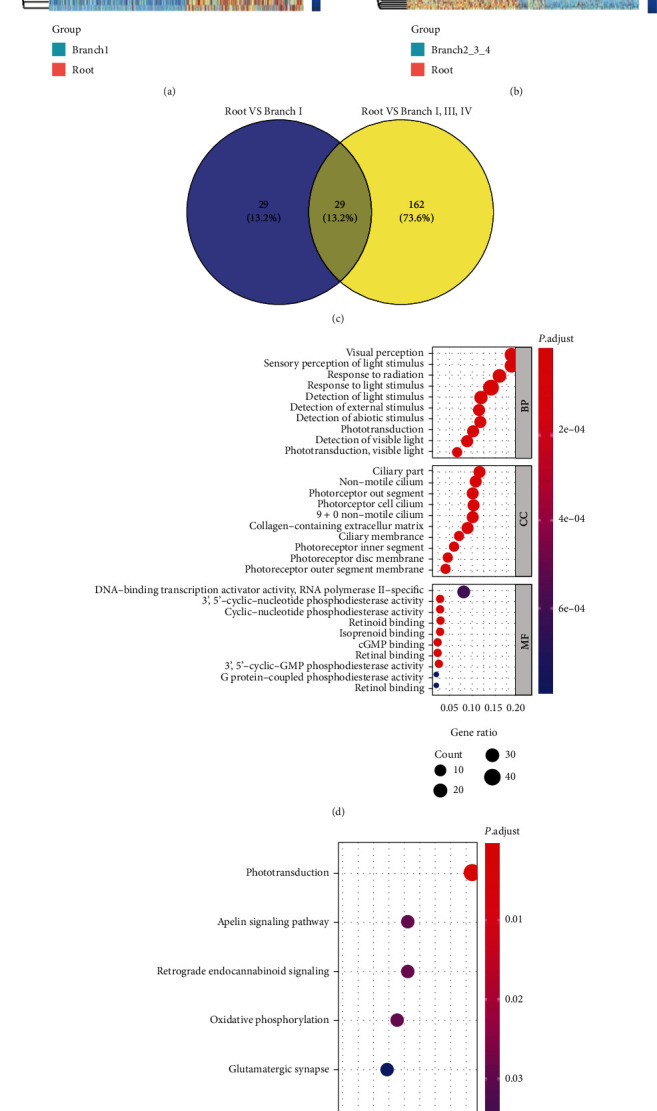
Identification of RDRGs and enrichment analyses. (a) Heat map of RDRGs obtained from differential analysis on root cells and branch I cells. (b) Heat map of RDRGs obtained from differential analysis on root cells and branch II, branch III, and branch IV cells. (c) Venn diagram of the two groups of RDRGs. 220 RDRGs are obtained by taking the union. (d) GO functional enrichment result of RDRGs. (e) KEGG pathway enrichment result of RDRGs.

Cells in the root region were taken as the control group, and 58 RDRGs were obtained by differential expression analysis with branch I cells (Figure 4(a)). Differential expression analysis was performed with branch II, branch III, and branch IV cells, and 191 RDRGs were obtained (Figure 4(b)). A total of 220 RDRGs were acquired by taking intersection with these RDRGs (Table S2) (Figure 4(c)). Further, GO enrichment analysis and KEGG pathway enrichment analysis were undertaken on these RDRGs. It was displayed that genes were significantly enriched in biological functions relevant to the vision and visual development, such as visual perception, sensory perception of light stimulus, response to radiation, response to light stimulus, eye development, visual system development, and sensory system development (Figure 4(d)). KEGG pathway enrichment analysis revealed that genes were mainly enriched in signaling pathways like phototransduction, apelin signaling pathway, retrograde endocannabinoid signaling, oxidative phosphorylation, and glutamatergic synapse (Figure 4(e)). On the whole, the 220 RDRGs were important when IPCSs differentiated into neurons and astrocytes and formed retina. Abnormal expression of these genes may also be relevant to degenerative retinal diseases.

### 3.4. Construction of PPI Network of RDRGs and Submodule Analysis

**Figure 5:**
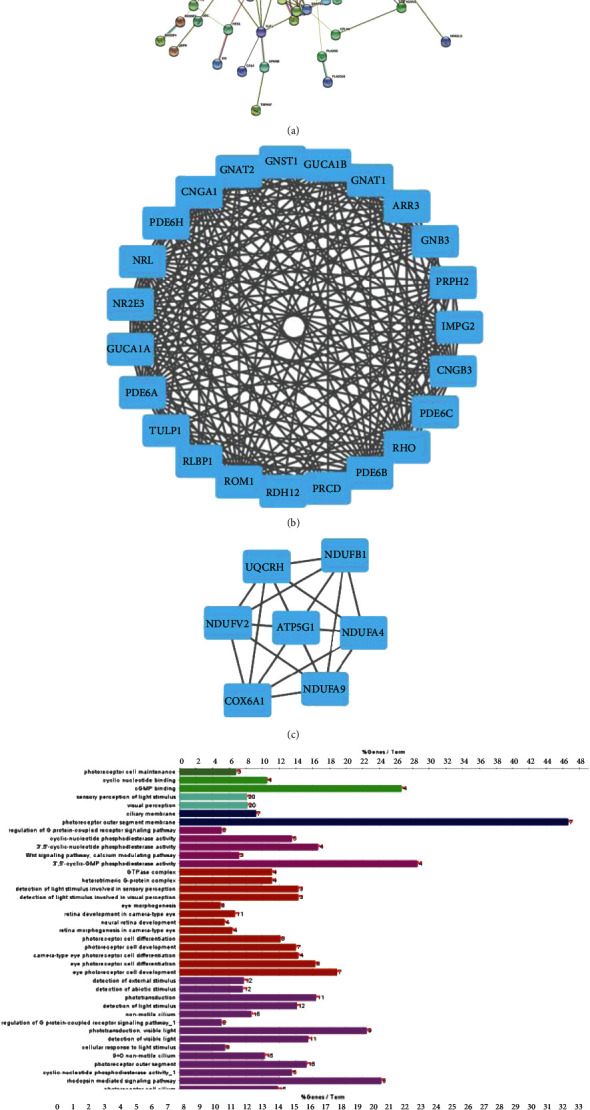
Construction of PPI network and functional submodule analysis. (a) PPI network constructed by RDRGs; nodes represent proteins, and connections represent protein-protein interactions predicted by STRING database. (b and c) Cluster 1 and Cluster 2 obtained by functional module analysis, respectively. (d and e) GO and KEGG enrichment analyses of Cluster 1 and Cluster 2, respectively.

PPI network of RDRGs was analyzed using STRING website, including 178 nodes and 864 interactions (Figure 5(a)). Clustering analysis was performed on PPI network via MCODE. We obtained functional submodule Cluster 1 containing 23 genes and Cluster 2 containing 7 genes (Figures 5(b) and 5(c)). ClueGO was used to conduct GO and KEGG enrichment analysis for the two submodules, and the results showed that Cluster 1 was mainly enriched in biological functions such as retinal development and visual perception and biological pathways of light transduction (Figure 5(d)). Cluster 2 was significantly enriched in respiratory-related biological functions, such as inner mitochondrial membrane complex, respirasome, and NADH dehydrogenase complex, and oxidative phosphorylation pathway (Figure 5(e)). There were two functional subsets in the PPI network of RDRGs. Genes in Cluster 1 were relevant to retinal development and visual signaling transduction. We speculated that aberrant expression of Cluster 1 genes may arise retinitis pigmentosa and degenerative retinal diseases.

## 4. Discussion

scRNA-Seq technology has gradually become the mainstream tool for cell heterogeneity investigation [18, 19]. Schafer et al. [20] performed scRNA-Seq on human cardiac fibroblasts and observed IL-11 as a critical factor for cardiovascular fibrosis. Der et al. [21] unraveled the molecular heterogeneity of lupus nephritis by scRNA-Seq. Guo et al. [22] applied scRNA-Seq to human embryonic stem cells and revealed bad effects of nicotine on the differentiation of these cells. Cells are the basic units of life activities, and understanding cell heterogeneity and differentiation is of medical value. In this study, bioinformatics methods were used to identify three cell populations that are important in the process of organoid differentiation. Through trajectory analysis, RDRGs were obtained, providing reference for the study of organoid differentiation mechanism and retinal diseases.

In the present study, dimension reduction analysis and cell type annotation were performed on scRNA-Seq data of human retinal organoids. Then, three cell populations during human retinal organoids differentiation were identified: iPSCs, neurons, and astrocytes. iPSCs are pluripotent stem cells obtained by reprogramming terminally differentiated somatic cells by artificially introducing specific transcription factors, which have similar morphology and function to embryonic stem cells [23]. The application prospect of these cells is broad in regenerative medicine, new drug development, and disease modeling [24, 25]. In the research of retinal diseases, Mandai et al. used iPSC generated in skin fibroblasts of a patient with neovascular age-related macular degeneration and induced iPSC to differentiate into retinal pigment epithelial (RPE). After one year of transplantation for the RPE sheet, the sheet structure is complete, and the optimal corrected visual acuity does not change [26]. Astrocytes are one of the 3 types of glial cells contained in human retina, and their presence and distribution are relevant to retinal blood vessels [27, 28]. Astrocytes show morphologic and functional changes in the early and late stages of almost every retinal vascular disease [29]. Guttenplan et al. [30] found that neurotoxic reactive astrocytes could produce neuronal death after retinal injury in a mouse glaucoma model. This suggests that astrocytes are the drivers of RGC death in chronic neurodegenerative eye diseases. Overall, this study used scRNA-Seq technology to identify three cell populations during differentiation of human retinoid organs. Our results are congruent with these reports, leading new directions for treatments of retinal diseases.

Trajectory analysis discovered two main differentiation features during retinal organoids formation: iPSCs differentiated into neurons and astrocytes. RDRGs were identified by gene differential expression analysis and then were subjected to enrichment analyses. GO functional annotation indicated that RDRGs were significantly enriched in biological functions relevant to the vision and visual development. KEGG pathway enrichment analysis showed that RDRGs were markedly enriched in pathways including phototransduction, apelin signaling pathway, retrograde endocannabinoid signaling, oxidative phosphorylation, and glutamatergic synapse. Chu et al. [31] found that apelin inhibited apoptosis of astrocytes and promoted angiogenesis. Feng et al. [32] also elaborated that apelin signaling pathway was critical to modulate vascular formation both pathologically and physiologically. In addition, oxidative phosphorylation is also associated with the vascular formation in retina-related diseases [33]. Endocannabinoid (eCB) is key to signaling transduction and synaptic function, and anterograde eCB participates in RGC to regulate CB1R-mediated calcium signaling [34]. Armed with the previous studies, it was posited that RDRGs screened in this study may be closely relevant to retinal development and retinal neural neuroregulation. 220 RDRGs we identified here may affect iPSCs differentiating into neuron and astrocyte cells. Aberrant expression of these genes may trigger degenerative retina diseases.

PPI network analysis is a commonly used method in bioinformatics analysis, and it systemically analyzed the interaction of massive proteins in the biologic system to reflect the role of genes in biological signaling transmission, substance metabolism, and cell cycle regulation under specific physiological status [35, 36]. Here, PPI network of RDRGs was analyzed through the STRING database, and then, two key submodules were obtained through subnetwork analysis. Enrichment analysis illuminated that Cluster 1 genes were mainly relevant to biologic functions of visual sensation like photoreceptor cell maintenance, photoreceptor cell differentiation, and photoreceptor cell development. Besides, KEGG enrichment analysis presented that Cluster 1 genes were significantly enriched in phototransduction signaling pathway. Phototransduction is a biological process of photoreceptor cells to transform the optical signal into an electrical signal. Opsins in photoreceptor cells can form photoreceptors with chromophore [37]. NRL and NR2E3 in Cluster 1 are pertinent to degenerative retina diseases. Rod differentiation factor NRL induces NR2E3 level to inhibit cone photoreceptor development [38]. NR2E3 and RHO are the genes enriched in PPI network. We speculated that NRL inhibited the expression of rod-specific genes such as NR2E3, thus inhibiting cone and rod development. A previous study found that light receptor phosphatase (PDE6) was a core rod and cone photoreceptor visual pathway in effecting enzyme, involved in visual signal transmission and amplification [39]. Rod PDE6 is also an essential heterodimer of PDE6A and PDE6B [40]. PDE6A mutation results in progressive retinitis pigmentosa [41]. Mutation of PDE6 family genes arose blockage of visual signal transmission, contributing to ophthalmic diseases, which was supported by KEGG results in this study.

Cluster 2 genes mainly function in mitochondrial energy supplying including NADH dehydrogenase complex and oxidative phosphorylation. Eyes are one of the most active organs in energy metabolism. An increasing amount of evidence supported that mitochondrial dysfunction was a cause of visual dysfunction [42, 43].

On the whole, this study acquired RDRGs through exploring scRNA-Seq data of human retinal organoids, showing that these genes were associated with retinal development, visual sensation, and signaling transduction. PPI network and functional submodule analyses indicated that RDRG-based PPI network encompassed Cluster 1 related to light transduction and Cluster 2 relevant to mitochondrial energy metabolism. The results in this study are hopeful to provide reliable theoretic evidence for studying retinal organoids and retina-related diseases. However, the present study is only based on bioinformatics analysis without any experimental proof. Further biochemical and clinical data are needed to support the results of this study. In addition, we only used one database for analysis, which reduced the reliability of experimental results. In future studies, we will collect more relevant samples and establish our own database to further verify our experimental results and increase the credibility of the results.

## Data Availability

The data used to support the findings of this study are included within the article. The data and materials in the current study are available from the corresponding author on reasonable request.

## Supplementary Materials

Supplementary Materials Data S1: sequencing data matrix of 1346 cells. Table S2: marker genes for each cell cluster. Table S3: retinal organoid cell differentiation-related genes.
