# Supplementary material for: Identification of Human Retinal Organoid Cell Differentiation-Related Genes via Single-Cell Sequencing Data Analysis
Source: Comput Math Methods Med. 2022 Aug 8;2022:9717599. doi: 10.1155/2022/9717599 (PMC9377943; doi:10.1155/2022/9717599)
Supplement: Supplementary Materials — Data S1: sequencing data matrix of 1346 cells. Table S2: marker genes for each cell cluster. Table S3: retinal organoid cell differentiation-related genes. [file 9717599.f1.zip › Table S2.pdf]

p_val	avg_logFC	pct.1	pct.2	cluster	gene
6.86E-52	1.277791	0.949	0.698	1	WIF1
6.63E-49	1.382381	1	0.978	1	TF
4.43E-48	0.934903	0.99	0.844	1	CLU
1.24E-46	0.884006	0.913	0.526	1	HES1
1.80E-45	1.167221	0.964	0.817	1	SPP1
5.19E-45	1.035922	0.964	0.722	1	DKK3
1.71E-44	0.933882	0.769	0.297	1	ADAMTS1
2.89E-43	1.026963	0.851	0.425	1	SLC1A3
1.05E-42	1.172797	0.887	0.54	1	CRYM
2.59E-39	0.946026	0.938	0.712	1	GLUL
3.92E-39	1.015513	0.841	0.434	1	RLBP1
1.73E-38	0.836399	0.974	0.753	1	VIM
6.61E-37	0.946246	0.974	0.786	1	CRYAB
4.76E-36	1.09531	0.877	0.567	1	FRZB
7.12E-36	0.889293	0.933	0.722	1	C1orf61
6.36E-34	0.945095	0.79	0.404	1	PRSS35
2.08E-33	0.980697	0.974	0.822	1	GPX3
4.39E-33	0.9047	0.718	0.317	1	SULF1
1.43E-31	0.846603	0.728	0.337	1	ZFP36L1
1.22E-30	0.884513	0.877	0.623	1	SAT1
4.64E-29	0.872029	0.779	0.431	1	PAX6
1.47E-26	0.868759	0.585	0.24	1	PLP1
5.57E-24	0.820093	0.79	0.486	1	GNG5
1.78E-23	0.854525	0.656	0.318	1	CCND2
4.77E-19	0.891349	0.646	0.351	1	SFRP2
1.01E-17	0.817948	0.631	0.341	1	RGS16
1.15E-08	-0.90437	0.882	0.922	1	PDE6G
7.75E-06	-0.94237	0.728	0.789	1	SAG
1.07E-05	-0.83014	0.713	0.765	1	GNAT1
2.19E-54	1.02962	0.977	0.488	2	GNAT2
6.75E-52	0.955234	1	0.934	2	GUCA1A
2.00E-50	1.08075	1	0.929	2	ARR3
3.62E-49	0.960865	1	0.968	2	PDE6H
1.15E-48	1.004045	1	0.734	2	GUCA1C
9.67E-46	0.998823	0.971	0.572	2	MYL4
3.26E-36	0.801569	0.869	0.4	2	MAP6
4.76E-31	0.850937	0.749	0.331	2	PLA2G5
4.85E-21	-1.52611	0.994	0.98	2	TF
8.98E-17	-1.10749	0.869	0.864	2	CLU
3.23E-15	-0.98116	0.834	0.829	2	TMSB4X
5.27E-13	-1.16995	0.766	0.788	2	VIM
5.69E-13	-1.52995	0.846	0.837	2	SPP1
8.81E-11	-1.31264	0.811	0.814	2	CRYAB
1.69E-10	-1.22648	0.771	0.755	2	DKK3
6.07E-09	-1.09612	0.349	0.501	2	PAX6
1.26E-08	-0.97858	0.863	0.841	2	GPX3
2.08E-08	-0.94033	0.343	0.508	2	SLC1A3
2.16E-08	-0.91027	0.623	0.687	2	GPM6B
3.33E-08	-1.29601	0.72	0.736	2	WIF1
4.61E-08	-1.26823	0.771	0.781	2	SAG
5.79E-08	-0.81753	0.514	0.593	2	HES1
6.63E-08	-0.99111	0.766	0.743	2	GNB1
1.67E-07	-0.97333	0.834	0.806	2	GNGT1
2.11E-07	-1.14851	0.794	0.746	2	C1orf61
2.30E-07	-0.86826	0.166	0.342	2	NES
2.51E-07	-1.03741	0.389	0.508	2	RLBP1
6.00E-07	-0.95044	0.646	0.689	2	CABP5

2.15E-06	-1.08113	0.749	0.758	2	GNAT1
7.22E-06	-0.93527	0.68	0.67	2	DBI
1.17E-05	-1.02891	0.474	0.556	2	IFITM3
2.35E-05	-0.83072	0.257	0.382	2	ADAMTS1
2.82E-05	-1.14305	0.531	0.566	2	PTN
3.11E-05	-1.26999	0.56	0.595	2	CRYM
4.48E-05	-1.06762	0.389	0.471	2	PRSS35
9.63E-05	-0.81463	0.943	0.868	2	ROM1
0.000158	-1.17746	0.52	0.581	2	RHO
0.000194	-0.80344	0.314	0.406	2	ZFP36L1
0.000288	-1.05832	0.617	0.611	2	FRZB
0.000293	-0.87411	0.314	0.412	2	IFITM2
0.000351	-0.88434	0.966	0.909	2	PDE6G
0.002512	-0.85313	0.32	0.392	2	RGS16
6.86E-45	1.126399	0.993	0.865	3	ROM1
1.29E-43	1.367485	0.986	0.756	3	SAG
1.50E-43	1.154842	0.979	0.718	3	GNB1
5.14E-42	1.133789	0.986	0.789	3	GNGT1
4.36E-39	1.238951	1	0.906	3	PDE6G
8.45E-38	1.215195	0.958	0.733	3	GNAT1
3.13E-32	0.964139	0.937	0.653	3	CABP5
1.54E-21	0.947264	0.783	0.537	3	REEP6
2.46E-21	0.908899	0.734	0.407	3	NR2E3
5.05E-20	0.803684	0.685	0.364	3	PDE6A
2.71E-16	0.934887	0.699	0.436	3	RASSF2
9.36E-16	-1.4131	0.615	0.787	3	GUCA1C
3.22E-15	-0.80779	0.273	0.608	3	SIX6
5.70E-15	-1.46846	0.93	0.939	3	ARR3
1.62E-13	-0.84594	0.531	0.761	3	CA2
1.71E-12	-0.91113	0.231	0.533	3	PCP4
3.48E-11	-1.01194	0.336	0.577	3	GNAT2
4.69E-11	0.916277	0.448	0.232	3	CCKBR
5.96E-11	-1.10382	0.965	0.973	3	PDE6H
6.45E-11	0.821089	0.301	0.116	3	CNGA1
1.78E-10	-0.9634	0.413	0.649	3	MYL4
1.66E-09	1.160034	0.685	0.559	3	RHO
2.60E-09	0.811448	0.455	0.264	3	SLC24A1
7.95E-08	-1.04802	0.622	0.747	3	WIF1
9.08E-08	-0.90357	0.42	0.635	3	FRZB
9.74E-08	-0.84301	0.916	0.946	3	GUCA1A
3.35E-07	-1.2174	0.748	0.849	3	SPP1
4.15E-07	-0.92896	0.664	0.831	3	CRYAB
7.21E-07	-0.81015	0.182	0.388	3	AKAP12
5.89E-06	-1.05129	0.455	0.607	3	CRYM
0.003713	-1.01165	0.965	0.983	3	TF
7.18E-49	1.295336	1	0.789	4	GNGT1
2.84E-42	1.135977	0.87	0.347	4	PDE6A
3.13E-42	1.196662	0.985	0.733	4	GNAT1
8.16E-42	1.338461	0.977	0.759	4	SAG
6.40E-39	1.02611	1	0.865	4	ROM1
1.33E-37	0.95187	0.985	0.72	4	GNB1
1.86E-37	0.944247	0.901	0.393	4	NR2E3
2.18E-37	0.860631	0.809	0.3	4	CLUL1
6.92E-37	1.109769	1	0.907	4	PDE6G
6.76E-34	0.938023	0.969	0.653	4	CABP5
1.64E-30	1.093322	0.641	0.213	4	CCKBR
5.62E-27	0.810902	0.664	0.244	4	SLC24A1
1.91E-22	1.327235	0.863	0.542	4	RHO

3.24E-09	-1.1789	0.931	0.939	4	ARR3
7.69E-09	-1.0951	0.687	0.778	4	GUCA1C
1.23E-07	-0.82625	0.076	0.286	4	PCDH9
3.95E-05	-0.81561	0.351	0.472	4	MAP6
9.66E-05	-0.93956	0.519	0.555	4	GNAT2
0.000112	-0.87616	0.588	0.628	4	MYL4
0.001304	-0.83077	0.748	0.733	4	WIF1
2.00E-57	0.804495	0.797	0.157	5	LITAF
2.52E-47	1.414266	0.983	0.503	5	IFITM3
2.77E-44	0.835068	0.729	0.176	5	TMEM37
2.30E-43	0.909569	0.644	0.14	5	S100A16
2.84E-42	0.833367	0.915	0.344	5	RHOC
6.14E-42	0.925542	0.678	0.164	5	NINJ1
6.10E-40	0.894462	0.788	0.238	5	F3
5.98E-39	1.066036	0.975	0.54	5	CNN3
1.59E-38	0.839837	0.831	0.272	5	ATP6V0E1
4.40E-38	1.11275	0.958	0.524	5	PTN
1.19E-37	0.845703	0.992	0.543	5	HES1
1.93E-37	0.844078	0.847	0.293	5	PET112
2.40E-37	1.109617	1	0.64	5	DBI
3.48E-37	0.809462	0.814	0.264	5	SPARC
4.38E-37	0.966908	0.941	0.443	5	SLC1A3
4.87E-37	1.15479	0.915	0.416	5	PRSS35
8.99E-37	1.313213	0.958	0.579	5	FRZB
1.13E-36	0.961045	0.975	0.487	5	GNG5
2.04E-36	1.199845	0.992	0.73	5	C1orf61
3.87E-36	1.371513	1	0.796	5	CRYAB
4.38E-35	0.981493	1	0.765	5	VIM
7.21E-35	1.173252	1	0.734	5	DKK3
2.43E-34	1.113281	0.847	0.357	5	IFITM2
3.28E-34	1.140289	1	0.852	5	CLU
4.87E-34	0.94326	0.975	0.65	5	GPM6B
6.23E-34	1.300568	0.712	0.227	5	LGALS1
7.29E-34	0.97109	0.644	0.178	5	LGALS3
1.08E-33	-1.383	0.983	0.963	5	GNB3
2.31E-32	1.160382	0.983	0.71	5	WIF1
9.47E-32	1.062649	0.966	0.63	5	SAT1
9.62E-32	0.897036	0.941	0.471	5	BTG2
1.05E-31	0.872291	0.797	0.297	5	DAPL1
1.25E-31	1.263808	0.992	0.823	5	SPP1
1.37E-31	1.360777	0.932	0.558	5	CRYM
4.89E-31	0.904422	0.475	0.102	5	ANXA2
2.96E-30	0.826116	1	0.836	5	FOS
3.54E-30	1.037055	0.814	0.341	5	RGS16
5.63E-30	0.898886	0.788	0.323	5	GADD45B
1.20E-29	1.164791	0.864	0.457	5	RLBP1
1.89E-29	0.909638	1	0.72	5	GLUL
5.65E-29	-1.04224	1	0.991	5	RCVRN
6.87E-29	1.047073	0.797	0.355	5	SFRP2
8.56E-29	0.877193	0.797	0.335	5	SULF1
1.07E-27	1.544451	0.839	0.392	5	IFITM1
2.46E-27	-0.9381	1	0.995	5	MAP1B
2.58E-27	0.874478	0.814	0.343	5	FABP7
1.52E-26	0.82489	0.941	0.572	5	IER2
2.13E-26	0.845675	0.847	0.428	5	RASSF4
3.21E-25	-1.09498	0.958	0.935	5	UNC119
6.78E-25	0.837027	0.72	0.281	5	ADM
1.97E-24	-1.49786	0.992	0.97	5	PDE6H

6.96E-24	-1.00115	0.941	0.938	5	AKAP9
1.56E-23	0.953628	0.712	0.286	5	ID3
3.32E-23	0.95913	0.458	0.121	5	IFIT2
3.34E-23	-1.26329	0.949	0.908	5	KCNV2
9.51E-23	1.167863	0.525	0.163	5	IFIT3
5.78E-21	-1.36107	0.941	0.892	5	GUCA1B
2.45E-20	1.083846	0.992	0.83	5	GPX3
1.42E-19	-1.1579	0.72	0.823	5	FSTL5
2.01E-19	-0.98062	0.907	0.864	5	ENO2
2.42E-19	-1.24793	0.89	0.887	5	04-Sep
1.02E-18	-1.40911	0.992	0.938	5	GUCA1A
1.03E-18	1.678533	0.805	0.453	5	ISG15
2.97E-18	-1.07655	0.746	0.833	5	NEUROD1
1.48E-17	-1.66376	0.975	0.935	5	ARR3
4.22E-17	1.345494	0.449	0.156	5	CCL2
1.08E-16	-1.09014	0.78	0.811	5	IMPG2
3.73E-15	-1.10807	0.89	0.85	5	CNGB1
1.15E-14	1.133376	1	0.98	5	TF
4.80E-14	-0.97158	0.729	0.793	5	PDC
1.03E-13	-0.90335	0.61	0.732	5	SNAP25
1.87E-13	-1.23329	0.703	0.741	5	GNGT2
3.66E-13	0.823281	0.415	0.157	5	AQP4
1.67E-12	-0.94832	0.907	0.846	5	AIPL1
1.36E-11	-0.87926	0.746	0.83	5	RP1
3.50E-11	-0.95911	1	0.91	5	GUK1
8.62E-11	-1.07558	0.898	0.876	5	ROM1
9.47E-11	-0.97428	0.686	0.731	5	SCG3
1.21E-10	-0.93197	0.636	0.727	5	PRPH2
1.26E-10	-0.91534	0.89	0.809	5	PLEKHB1
1.84E-10	-1.04606	0.525	0.663	5	IMPG1
2.34E-10	-0.90697	0.297	0.542	5	LOC157627
9.11E-10	-0.90919	0.593	0.674	5	CPLX4
9.14E-10	-0.88251	0.517	0.685	5	RBP3
2.91E-09	-1.08283	0.525	0.647	5	ABCA4
2.93E-09	-0.94368	0.593	0.676	5	CHN2
5.46E-09	-1.05796	0.602	0.669	5	RS1
1.20E-08	-0.94313	0.686	0.688	5	VTN
1.29E-08	-1.0365	0.585	0.658	5	CC2D2A
1.39E-08	-1.27562	0.839	0.762	5	GUCA1C
3.09E-08	-0.86431	0.61	0.686	5	PROM1
1.21E-07	-0.89042	0.703	0.725	5	AHI1
1.32E-07	-0.84667	0.508	0.61	5	STX3
3.96E-07	-0.90933	0.958	0.912	5	PDE6G
4.95E-07	-0.82485	0.449	0.562	5	MPP4
2.42E-06	-1.40282	0.661	0.621	5	MYL4
3.55E-06	-1.04012	0.475	0.559	5	GNAT2
6.11E-06	-0.81653	0.898	0.779	5	LBH
1.58E-05	-0.90378	0.39	0.511	5	PCP4
5.88E-05	-0.85148	0.475	0.534	5	RGS9
8.06E-05	-0.93528	0.373	0.468	5	LMOD1
9.23E-05	-0.81555	0.517	0.574	5	CDHR1
0.009794	-0.81653	0.805	0.752	5	GNAT1
1.68E-48	1.169499	1	0.969	6	PDE6H
2.89E-48	1.237131	1	0.748	6	GUCA1C
1.36E-46	1.321326	1	0.933	6	ARR3
5.25E-44	1.134427	1	0.59	6	MYL4
7.94E-43	1.032254	1	0.51	6	GNAT2
5.99E-40	0.971999	1	0.938	6	GUCA1A

4.07E-39	0.934104	1	0.911	6	GUK1
1.88E-37	0.832662	0.929	0.342	6	HRASLS
3.12E-37	0.95088	0.965	0.414	6	MAP6
6.51E-37	0.949771	1	0.714	6	GNGT2
1.15E-33	0.826977	0.938	0.416	6	LMOD1
1.47E-32	0.97145	0.832	0.345	6	PLA2G5
1.33E-27	0.825894	0.735	0.261	6	TTR
6.16E-22	-1.91621	0.991	0.981	6	TF
4.32E-14	-1.29309	0.965	0.856	6	CLU
7.59E-13	-1.05105	0.823	0.83	6	TMSB4X
4.28E-09	-1.69303	0.867	0.835	6	SPP1
6.28E-09	-1.48366	0.894	0.806	6	CRYAB
4.79E-08	-1.20983	0.823	0.782	6	VIM
4.92E-08	-1.11948	0.956	0.834	6	GPX3
1.16E-07	-1.03648	0.805	0.739	6	GLUL
1.96E-07	-1.5012	0.779	0.73	6	WIF1
4.42E-06	-1.29144	0.832	0.75	6	DKK3
5.53E-06	-1.0336	0.876	0.804	6	GNGT1
7.28E-06	-1.37217	0.876	0.741	6	C1orf61
1.04E-05	-1.43721	0.867	0.772	6	SAG
1.09E-05	-1.06264	0.699	0.682	6	CABP5
4.49E-05	-1.01834	0.991	0.909	6	PDE6G
0.000142	-0.90405	0.973	0.869	6	ROM1
0.000192	-1.2868	0.894	0.745	6	GNAT1
0.000315	-0.84646	0.265	0.376	6	CCND2
0.000374	-1.0829	0.319	0.406	6	PDE6A
0.000771	-0.86729	0.274	0.374	6	ADAMTS1
0.000801	-0.89331	0.319	0.401	6	ZFP36L1
0.001396	-1.04696	0.407	0.465	6	PRSS35
0.00162	-1.1789	0.575	0.56	6	PTN
0.002537	-1.37987	0.566	0.573	6	RHO
0.003827	-1.38542	0.646	0.586	6	CRYM
0.004914	-0.89044	0.903	0.732	6	GNB1
0.005377	-1.12009	0.575	0.543	6	IFITM3
0.006125	-1.00043	0.496	0.486	6	SLC1A3
0.009805	-0.89685	0.336	0.379	6	SULF1
1.75E-37	0.958805	0.396	0.052	7	TAGLN3
4.15E-34	1.414364	0.368	0.051	7	TMEM215
5.02E-31	1.490163	0.406	0.073	7	STMN2
1.97E-28	1.6976	0.425	0.089	7	ISL1
1.99E-28	0.822703	0.274	0.031	7	GABRR1
5.03E-24	0.88249	0.292	0.046	7	PCP4L1
3.23E-19	0.986339	0.358	0.088	7	CA10
3.80E-17	0.8543	0.953	0.89	7	SOX4
7.45E-16	0.833131	0.642	0.329	7	CELF4
4.19E-15	1.277036	0.519	0.215	7	CPLX3
6.18E-13	-0.80539	0.84	0.918	7	KCNV2
7.27E-13	0.805342	0.726	0.476	7	CCDC88A
9.66E-12	1.235863	0.302	0.098	7	ONECUT2
1.99E-11	1.095061	0.16	0.03	7	LRTM1
7.91E-11	0.884948	0.425	0.192	7	SERPINI1
1.05E-10	0.843122	0.358	0.144	7	RTN1
1.36E-10	-1.13064	0.981	0.971	7	PDE6H
1.63E-10	-1.33278	0.934	0.939	7	ARR3
2.03E-10	-0.97835	0.943	0.943	7	GUCA1A
2.28E-10	0.851538	0.217	0.058	7	SEMA3E
7.96E-10	0.873505	0.34	0.129	7	ARID5B
8.47E-10	-0.8143	0.887	0.897	7	GUCA1B

1.38E-09	-1.31701	0.443	0.64	7	MYL4
3.08E-09	1.167903	0.179	0.046	7	CXCL14
5.99E-08	0.924314	0.547	0.327	7	CADPS
9.20E-08	-0.82774	0.349	0.569	7	GNAT2
3.61E-07	-1.16314	0.726	0.773	7	GUCA1C
1.69E-06	-0.83344	0.198	0.408	7	HRASLS
0.000164	-0.80149	0.443	0.603	7	CRYM
0.005354	0.99507	0.321	0.236	7	CNTNAP2
9.28E-29	0.925794	1	0.939	8	GUCA1A
2.36E-27	0.802207	1	0.962	8	GNB3
2.74E-27	0.95069	1	0.97	8	PDE6H
1.83E-25	0.985799	1	0.934	8	ARR3
5.95E-25	0.965413	0.988	0.754	8	GUCA1C
1.34E-20	0.920324	0.953	0.602	8	MYL4
4.22E-11	-1.0775	0.721	0.837	8	TMSB4X
2.21E-10	-1.51101	0.988	0.981	8	TF
1.90E-09	-1.09004	0.779	0.871	8	CLU
4.92E-09	-1.12243	0.256	0.583	8	PTN
1.44E-08	-1.17529	0.628	0.822	8	GNGT1
1.94E-08	-0.86676	0.326	0.6	8	HES1
2.30E-08	-1.09074	0.628	0.796	8	VIM
3.45E-07	-1.31756	0.419	0.625	8	FRZB
5.86E-07	-1.11579	0.547	0.747	8	WIF1
1.77E-06	-1.3942	0.791	0.841	8	SPP1
1.31E-05	-0.83981	0.058	0.268	8	CCKBR
1.52E-05	-0.95711	0.291	0.494	8	PAX6
1.79E-05	-1.05972	0.663	0.788	8	SAG
2.59E-05	-1.20183	0.779	0.816	8	CRYAB
2.62E-05	-0.90558	0.628	0.766	8	DKK3
2.76E-05	-0.82471	0.43	0.583	8	TMSB10
4.11E-05	-0.91865	0.209	0.411	8	PDE6A
9.19E-05	-0.91695	0.36	0.558	8	IFITM3
0.000128	-0.94911	0.198	0.395	8	RGS16
0.00014	-1.20865	0.453	0.6	8	CRYM
0.000154	-0.94503	0.326	0.504	8	RLBP1
0.000166	-0.81439	0.547	0.68	8	DBI
0.000255	-0.80854	0.221	0.392	8	LOC645323
0.000481	-0.93618	0.686	0.757	8	C1orf61
0.001075	-1.07694	0.721	0.76	8	GNAT1
0.00228	-0.96569	0.802	0.847	8	GPX3
