# Supplementary material for: Identification of Human Retinal Organoid Cell Differentiation-Related Genes via Single-Cell Sequencing Data Analysis
Source: Comput Math Methods Med. 2022 Aug 8;2022:9717599. doi: 10.1155/2022/9717599 (PMC9377943; doi:10.1155/2022/9717599)
Supplement: Supplementary Materials — Data S1: sequencing data matrix of 1346 cells. Table S2: marker genes for each cell cluster. Table S3: retinal organoid cell differentiation-related genes. [file 9717599.f1.zip › Table S3.pdf]

gene
ARR3
GUCA1C
PDE6H
GUCA1A
KCNV2
GNB3
GUCA1B
GNAT2
MAP1B
CLU
MYL4
TF
GUK1
GNGT2
CC2D2A
UNC119
RS1
RCVRN
SPP1
PDC
CNGB3
LBH
AKAP9
04-Sep
ACTB
IMPG2
WIF1
PRCD
SCG3
IMPG1
ENO2
VIM
HES1
ACTG1
CRYAB
MAP6
DKK3
FSTL5
TULP1
AHI1
LMOD1
VTN
NEUROD1
PLEKHB1
CHN2
NDUFA4
GPX3
PDE6C
RABL3
HRASLS
C1orf61
CPLX4
COX6A1
SNAP25
RGS9
SLC1A3
ARL6IP5

TMSB4X
MATN2
FOS
ITM2C
UQCRH
XRCC4
PLA2G5
PRDX1
ELOVL4
PRPH2
SERPINF1
ADAMTS1
NDUFB1
GLUL
FRZB
PPA1
TSPAN7
PAX6
RAB41
RLBP1
PCP4
KIF2A
CRABP1
PRSS35
NDUFA9
ZFP36L1
PTN
RDH12
SOX4
NDUFV2
PDE6D
GPM6B
ATP5G1
SULF1
F3
CRYM
TMEM14B
FTH1
COL2A1
IFITM3
SAT1
NR4A1
DHRS7
FOSB
CCND2
TTR
GPR37
DBI
TMSB10
EGR3
ENO1
CYR61
SOX2
MGST3
RBP4
ATF3
LAPTM4A
ALDOC

PLP1
TUBA4A
ZFP36
CD9
NES
LITAF
GNG5
LIX1
RGS16
CNN3
SOX9
TMEM37
PON2
AKAP12
SFRP2
RHOC
CFI
RNF113A
LAP3
RASSF4
MCL1
LGALS3BP
LGALS1
CA14
SPARC
CCND1
IFITM2
SH3BGRL
TRAPPC5
TUBB2B
GADD45B
TMEM98
FXYD6
FABP7
RGR
SPINK4
EGR1
SERPING1
S100A16
QDPR
RDH10
IER2
FEZ1
MFAP4
OAF
ATP6V0E1
C1orf54
LGALS3
ID3
BTG2
RBP1
FXYD3
TSPAN6
RARRES2
MAGED2
PLA2G16
NDP
FABP5

ANXA5
UBE2L6
DAPL1
NGFRAP1
TNFRSF12A
MARCKSL1
ISG15
IFITM1
H1F0
ADORA2B
IFIT2
BST2
NUPR1
NINJ1
S100A13
KLF10
ADM
CSRP2
ROM1
GNGT1
PDE6G
SAG
GNB1
CABP5
GNAT1
CADM1
SPTBN1
SIX6
NR2E3
CA2
PDE6A
KCNB2
CLUL1
DPCD
REEP6
RHO
CCKBR
DDC
KLF6
RASSF2
NRL
GSTP1
LEMD1
PDE6B
CNGA1
PDCD4
PPAP2C
